# The impact of information sources on COVID-19 vaccine hesitancy and resistance in sub-Saharan Africa

**DOI:** 10.1186/s12889-022-14972-2

**Published:** 2023-01-06

**Authors:** Uchechukwu L. Osuagwu, Khathutshelo P. Mashige, Godwin Ovenseri-Ogbomo, Esther Awazzi Envuladu, Emmanuel Kwasi Abu, Chundung Asabe Miner, Chikasirimobi G. Timothy, Bernadine N. Ekpenyong, Raymond Langsi, Onyekachukwu M. Amiebenomo, Richard Oloruntoba, Piwuna Christopher Goson, Deborah Donald Charwe, Tanko Ishaya, Kingsley E. Agho

**Affiliations:** 1 Bathurst Rural Clinical School (BRCS), School of Medicine, Western Sydney University Bathurst, Bathurst, NSW 2795 Australia; 2 African Vision Research Institute, Discipline of Optometry, Westville Campus, University of KwaZulu-Natal, Durban, 3629 South Africa; 3 Translational Health Research Institute (THRI), Western Sydney University, Campbeltown, NSW 2560 Australia; 4 Department of Optometry, University of the Highlands and Islands, Inverness, IV2 3JH UK; 5 Department of Community Medicine, College of Health Sciences, University of Jos, Jos, 930003 Nigeria; 6 Department of Optometry and Vision Science, School of Allied Health Sciences, College of Health and Allied Sciences, University of Cape Coast, Cape Coast, 00233 Ghana; 7 Department of Community Medicine, College of Health Sciences, University of Jos, Jos, 930003 Nigeria; 8 Department of Optometry, Faculty of Health sciences, Mzuzu University, P. Bag 201 Luwinga 2,, Mzuzu, Malawi; 9 Department of Public Health, Faculty of Allied Medical Sciences, College of Medical Sciences, University of Calabar, Cross River State, Calabar, 540271 Nigeria; 10 Health Division, University of Bamenda, Bambili, P. O. Box 39, Cameroon; 11 Department of Optometry, Faculty of Life Sciences, University of Benin, Benin, Nigeria; 12 School of Optometry and Vision Sciences, College of Biomedical Sciences, Cardiff, CF24 4HQ UK; 13 School of Management and Marketing, Curtin Business School, Curtin University, Bentley, WA 6151 Australia; 14 Department of Psychiatry, College of Health Sciences, University of Jos, Jos, Nigeria; 15 Tanzania Food and Nutrition Center, P. O. Box 977, Dar es Salaam, Tanzania; 16 Department of Computer Science, University of Jos, Jos, 930003 Nigeria; 17 School of Health Sciences, Western Sydney University, Campbelltown, NSW 2560 Australia

**Keywords:** Coronavirus, Facebook, Media, Africa, Television, Misinformation, Survey, Radio, Healthcare workers, Lockdown

## Abstract

**Background:**

Vaccination remains the most powerful weapon against the emergence of new variants of coronavirus (COVID-19). However, false information about COVID-19 vaccines through various platforms including social media remains a major threat to global public health. This study examined the impact of information sources on COVID-19 vaccine hesitancy and resistance in sub-Saharan Africa (SSA).

**Methods:**

A validated web-based cross-sectional study was conducted from 14 March to 16 May 2021, and was administered in both French and English to 2572 participants aged 18 years and over. Data on sociodemographic characteristics, medical and vaccination history, and the information sources (mainstream media and social media) used by the participants during the pandemic were obtained. There were three main outcomes: The vaccinated group were those who responded in the affirmation (Yes) to the question of whether they have been vaccinated against COVID-19. Those who responded ‘not sure’ or ‘no’ to the question were then asked if they were willing to be vaccinated when the vaccine became available in their home countries. The responses to this follow-up question were used to derive the second and third outcome variables of ‘vaccine hesitancy’ and ‘vaccine resistance’, respectively. A series of logistic regression analyses were used to examine the impact of information sources on the three main outcomes.

**Results:**

The prevalence of COVID-19 vaccine hesitancy among the participants was lowest among newspaper readers (42%) and highest among TV (72%) and social media users (73%). The prevalence of COVID-19 vaccine-resistance was also lowest among newspaper readers (37%) but highest among social media users (87%). Multivariate analyses revealed that compared to those who did not use these information sources, SSA participants who relied on the radio (aOR 0.83, 95%CI = 0.70, 0.99), TV (aOR 0.80, 95%CI = 0.65, 0.97) and social media (aOR 0.79, 95%CI = 0.65, 0.97) for information during the pandemic were less likely to be hesitant towards taking the vaccines. However, social media users (aOR 2.13, 95%CI = 1.62, 2.80), those who watched TV (aOR 1.40, 95%CI =1.08, 1.80), relied on healthcare workers (HCWs: aOR 1.32, 95%CI = 1.07, 1.63) and families/friends (aOR 1.31, 95%CI = 1.06, 1.61) for COVID-19 related information during the pandemic were more likely to resist taking the COVID vaccines in this study. Participants who relied on the newspaper for information during the pandemic were less likely to resist the vaccines (aOR 0.77, 95%CI = 0.62, 0.95) compared to non-readers of a newspaper.

**Conclusion:**

We found that all six information sources except radio were strong predictors of the resistance towards COVID-19 vaccination. Further research on how these channels can be used to improve the availability of reliable healthcare information is needed. Investments in these resources will protect people and empower them to make appropriate choices about their health.

**Supplementary Information:**

The online version contains supplementary material available at 10.1186/s12889-022-14972-2.

## Background

The COVID-19 pandemic has significantly impacted economic, health and living conditions on the African continent and elsewhere [1, 2]. The impact on individuals, families and communities across Africa has been unprecedented. While the global economic loss is still unfolding, it is projected to be quite huge particularly in African countries [3]. The risk of COVID-19 resurgence remains high in several African countries due to poor adherence to public health measures, mass gatherings, low testing and low vaccination rates [4]. This resurgence creates more demands on an already depleted and struggling healthcare system thereby leaving many of the citizens in a dilemma. Governments are also overburdened with balancing the provision of care regarding the presence of other viral infections and diseases that have sprung up again due to all attention being diverted to the COVID-19 pandemic as is seen in countries like the Democratic Republic of Congo (Ebola), Lassa fever in Guinea, Liberia, Kenya (Rift valley Fever), Nigeria and Sierra Leone, Republic of Guinea (Marburg virus disease), among other African countries [5–8]. Furthermore, residents have purchased and stored some medications commonly used for treating other infectious diseases causing scarcity, and rising costs due to an increase in demand [9].

Vaccination remains the most powerful weapon against the emergence of new variants [10] as well as reaching herd immunity [11]. However, compared with the rich European and North-American countries, COVID-19 vaccination remains very low among African countries with only 11% of the adult population fully vaccinated [10]. This lack of adequate and complete vaccination of the populace, among other factors, is brought about by the state of the economy in African countries. Most African countries are in the low-middle income strata. High income economies, purchase and hoard vaccines immediately or even before they are mass produced by paying pharmaceutical companies huge deposits for these vaccines before production which affects the vaccine distribution globally. This also limits effective control of the widely spreading disease, particularly among African countries and thus the emergence of various variants of the virus as seen in South Africa (omicron), Brazil (delta) and India [12]. This act of hoarding vaccines could be directly attributable to the non-achievement of disease control and its resurgence in other variants in low-middle-income countries. As such the inability to attain community immunity globally since people are still travelling, more so, with most of these countries lowering their guard on the earlier preventive measures [12].

The African continent has witnessed four waves of COVID-19 over the last 2 years and has improved its capacity to manage COVID-19 cases [10]. The supply of COVID-19 vaccines across the region has also increased with approximately 672 million doses distributed across the region, mostly facilitated by COVAX (65%) and the rest through bilateral deals (29%) and the African Union’s Vaccines Acquisition. Despite this improvement, there are concerns that the rapid spread of ‘false or misleading information’ in digital and physical environments causes confusion and risk-taking behaviours that can harm health and lead to mistrust in health authorities and undermine the public health response [13]. For instance, in Pakistan, vaccine hesitance and resistance fuelled by fear of the unknown, country of manufacture of the vaccine, religious and cultural ideologies, have made it almost impossible to reach the people [14]. Yet, despite the widespread concern about the potential impacts of misinformation on vaccination, little is known about the magnitudes of those impacts nor their differential effects across various countries in sub-Saharan Africa (SSA).

Exposure time to COVID-19-related news increased over time during the pandemic [15] and more exposures to the news have direct implications on people’s actions such that receiving timely and informative communication during a time of uncertainties promotes public cooperation [16]. Infodemic affects the hesitance and resistance to uptake of new products across the market, and it becomes worse in a pandemic as seen with the coronavirus disease and its management and supposed consequences [13]. Vaccine hesitancy (reluctance to receive vaccines) is one of the top ten threats to global health [17] and this is fuelled by health information obtained from the news media, internet and social media platforms [18–21]. Vaccine hesitancy is also high among certain population groups [22, 23] probably due to the previous medical experiment amongst these population groups [24] and poor messaging [25]. Misinformation regarding the benefits, medicinal composition, and adverse effects of vaccination, limits patient understanding and overall buy-in [18]. Although access to technology has improved during the pandemic, and the use of social media has increased [18], there are concerns about the spread of misinformation across different social networks propagated via the contemporary anti-vaccination movement, to fuel vaccine hesitancy [26, 27]. This has the potential to compromise public confidence in the COVID-19 vaccine for the prevention of the disease [28]. However, where social media platforms were used to propagate healthy messages, by nurses and doctors, a significant improvement in compliance with public health messages and subsequent COVID-19 infections has been reported [21].

Sources of vaccination information have different effects on people’s coping appraisal of COVID-19 vaccination [20]. Unlike mainstream media, social media such as Facebook, Twitter, Instagram, WhatsApp, and Pinterest allow individuals to rapidly create and share content globally without editorial oversight [29, 30]. These are complex and fluid ecosystems, in which anti-vaccination viewpoints can be amplified and represented as mainstream, and vaccine-hesitant parents can encounter compelling narratives from other parents dissuading vaccination [31]. Misinformation and unsubstantiated rumours regarding COVID-19 and potential vaccination against SARS-CoV-2 have already begun emerging on social media platforms, threatening to erode public confidence as the vaccines are rolled out in African countries [32]. Information spread through social media directly or indirectly increases hesitancy toward COVID-19 vaccination, while the opposite effect was observed for institutional websites [27]. Since social media platforms may self-select content streams, contributing to ideological isolation, owners must ensure that social media platforms provide access to accurate information on the safety and efficacy of vaccinations [29].

The uptake of COVID-19 vaccination in SSA may be impeded by the rapid spread of misinformation on social media leading to belief in false rumours about the pandemic [29], which has been associated with poor health-seeking behaviour [33, 34]. The recent mixed international messages about the efficacy of the different COVID-19 vaccines, their side effects beyond the local and systemic effects [35, 36] and the lack of clarity regarding the required dosage [37] may further reduce the confidence of African populations in the safety of the vaccines [21]. In addition, the halting of the AstraZeneca vaccine in South Africa, which showed less protection against the new variant SARS-CoV-2 that can evade key antibodies [21], may have contributed to lower people’s confidence in the vaccine efficacy. Healthcare workers are among the most trusted experts [38–40].

Intensive global efforts for continued physical distancing and isolation to curb the spread of new strains of SARS-CoV-2 may intensify the use of social media as individuals try to remain connected while apart [41]. In a randomized controlled trial to understand the impact of social media in the United States, researchers found that messages spread by nurses and doctors on social media led to a significant reduction in holiday travel and subsequent COVID-19 infections [21]. Therefore, identifying, understanding, and addressing how information sources affect vaccine acceptance [42], hesitancy and resistance [43] is potentially important to increase vaccine uptake.

Therefore, this study was designed to, a) determine the proportions of SSA participants that were dependent on the different sources of information (social media and mainstream media sources) for COVID-19-related information; b), profile individuals who use the mainstream media outlets (TV and radio, newspaper) to obtain COVID-19 related information by identifying the key socio-demographic, and health-related factors that are associated with the different information sources; and c), determine the sources of information about the COVID-19 pandemic among vaccine-hesitant and resistant individuals across SSA countries as well as identify the association between sources of information and vaccine hesitancy. By identifying the distinguishing characteristics, public health officials may be better able to target a sub-population at greater risk of exposure to misinformation about the COVID-19 vaccine. Findings will also offer a greater understanding of how public health officials can effectively tailor health behaviour messaging to align with the socio-demographic profiles of vaccine-hesitant or resistant individuals, while also considering their consumption of COVID-19 information and the predominant sources. In addition, the study findings will help to provide steps on how social media may be used to improve health literacy and build public trust in vaccination.

## Materials and methods

### Survey design

This was a cross-sectional study that recruited participants across SSA countries between March 14 and May 16, 2021. The questionnaire was initially developed and used for a similar study [44]. The questionnaire was tested for the internal validity of the items, and Cronbach’s alpha coefficient score ranged from 0.70 and 0.74, indicating satisfactory consistency [44]. The questionnaire was adapted with minor modifications to suit this study’s objective and was made available in English and French languages to allow for residents residing in the Anglophone and Francophone SSA countries to participate. This was also necessary to increase the reach of the survey, one of the past study limitations [33, 34]. Moreover, a pilot study was conducted on 10 participants who were not included in the final study and were not part of the research group to ensure clarity and understanding as well as to determine the duration of completing the questionnaire before dissemination. The final questionnaire is presented as Supplementary Table S1.

### Participants

Eligible participants were adults of SSA origin, living in or outside of Africa, aged 18 years and older, who were able to provide informed consent at the time of this study. Since this was an online survey, it is possible that participants were those who had access to the internet and those who were on their respective social media platforms and used them. Participants were excluded if they were not from SSA countries, were younger than 18 years, were unable to provide informed consent, and participated in the initial pilot study. The supplementary Fig. S1 shows the distribution of the participants by their countries of origin.

Using a snowball sampling technique, participants were recruited online after the survey was created in survey monkey (SurveyMonkey Inc., San Mateo, California, USA, www.surveymonkey.com) and was administered in two languages. An e-link to the survey was disseminated via emails and posted on social media platforms (Facebook and WhatsApp). The distribution of the survey was strongly reliant on the snowballing or chain-referral approach using virtual networks to reach the population who used social media and other online formats, thus saving time and cost for data collection [45, 46]. Authors were also encouraged to share the e-link of the survey through personal emails and social network groups in their respective countries. The use of an online survey ensured that a large spectrum of prospective participants across SSA could be reached in limited time and resources.

The sample size calculation was based on a single population proportion formula by the World Health Organization (WHO) as well as previous studies [33, 34, 47]. Assuming a 20% attrition rate for a proportion of 50% of the population and using the desired precision of 2% and the 5% significance level for a two-sided test to detect statistical differences between groups at 80% power, a sample size of 2502 was considered adequate for this study aims.

### Dependent variables

The main outcomes were the three COVID-19 vaccine indicators of the participants. The vaccinated group was formed by those who responded in the affirmation (Yes) to the question of whether they have been vaccinated against COVID-19. Those who responded ‘not sure’ or ‘no’ to the question were then asked if they were willing to be vaccinated when the vaccine became available in their home countries. The responses to these follow-up questions were used to derive the second and third outcome variables of ‘vaccine hesitancy’ and ‘vaccine resistance’, respectively, similar to a previous study [48]. In this study, vaccine acceptance refers to a position ranging from passive acceptance to active demand [42], whereas hesitancy and resistance, respectively, were used to define the reluctance to receive vaccines (i.e. positions of being unsure about taking a vaccine) and being absolutely against taking a vaccine [43].

### Exposure variables

The exposure variables were derived from the question of how the participants obtained information on the COVID-19 vaccine. The participants responded ‘yes’ or ‘no’ to whether they obtained the information from the mainstream media (Radio, Television, Newspaper), Social media (such as Facebook, WhatsApp, Twitter) or healthcare workers (HCWs), or family and friends.

### Independent variables

The questionnaire included demographic data (age group, sex, country of origin, religion, marital status, educational level, employment status, occupational status), health indicator factors (smoking status, presence of pre-existing conditions including diabetes, lung disease, heart disease, hypertension, obesity, asthma) and previous immunisations/vaccines history. These constituted the independent variables.

### Statistical analysis

Analyses were performed using STATA/MP version 14 (Stata Corp, College Station, TX, USA) and categorical data are shown as counts and percentages. The proportion of participants who used each of the sources of information was conducted using cross-tabulation. The proportion of participants who used each of the sources of information was conducted using cross-tabulation. The associations between sources of information and vaccine hesitancy and resistance were determined in a series of logistic regression analyses that included sources of information as exposure variables after controlling for demographic factors, and health indicator factors. There is no unique statistical test for multicollinearity for binary logistic regression but in our analysis, we treat the binary outcome variables as a continuous variable and used the “Logit” command and then ‘collin’ command in Stata to determine multicollinearity including Variance Inflation Factors (VIF) because collinearity is driven by the characteristic of the independent variables and no the type of regression used [49] and the VIF < 4 was considered suitable [50]. The odds ratios with 95% confidence intervals (CI) were calculated to assess the adjusted odds of exposure and independence variables.

### Ethical consideration

This self-administered web-based cross-sectional study was approved by the Humanities and Social Sciences Research Ethics Committee (HSSREC 00002504/2021) of the University of KwaZulu-Natal, Durban, South Africa. The study adhered to the principles of the 1967 Helsinki declaration (as modified in Fortaleza 2013) for research involving human subjects. Before the study, an explanation detailing the nature and purpose of the study was provided to all participants using an online preamble. Informed consent was obtained from the participants who were required to answer either a ‘yes’ or ‘no’ to a question on whether they were willing to voluntarily participate in the survey. The confidentiality of participant responses was assured, and anonymity was maintained. Participation in the study was voluntary without any incentive, inducement, or obligation from the researchers. To ensure that only one response per participant was included in the study, participants were instructed not to take part in the survey more than once, and during analysis, we also restricted the data by the IP address of the participants.

## Results

The socio-demographic characteristics of the 2572 participants who took part in this study are reported in Table 1. Of these participants, 1390 were males (54%), mostly educated (80% of the participants had completed a bachelor’s or higher education degree), about one-third were aged 18-28 years (929, 36.1%), and more than half of them were not married (1440, 56.0%) and resided in West African countries (1446, 56.2%). About 80% of the participants were employed in non-healthcare sectors and of health indicators, there were few smokers (177, 6.9%) and people who reported that they had a pre-existing condition (880, 34.2%).

**Table 1:** Distribution (n, %) of the socio-demographic characteristics of the participants and their main sources of COVID-19 related information during the pandemic

Variables	All	Radio	TV	Newspaper	Social media	HCW	Family/friends
**n, %**	2572 (100)	1449 (56.3)	1897(73.8)	1067 (41.5)	1879 (73.1)	1289 (50.1)	1215 (47.2)
**Demography**							
**Age category in years**^a^							
18-28	929 (36.1)	497 (54.0)	656 (70.6)	347 (37.4)	682 (73.4)	437 (47.0)	461 (49.6)
29-38	720 (28.0)	415 (57.6)	532 (73.9)	293 (40.7)	523 (72.6)	363 (50.4)	321 (44.6)
39-48	502 (19.5)	293 (58.4)	390 (77.7)	212 (42.2)	364 (72.5)	271 (54.0)	228 (45.4)
49+	346 (13.5)	201 (58.1)	271 (78.3)	177 (51.2)	265 (76.6)	178 (51.4)	164 (47.4)
**Sex**							
Males	1390 (54.0)	829 (59.6)	1047 (75.3)	629 (45.2)	1028 (74.0)	690 (49.6)	623 (44.8)
Females	1182 (46.0)	620 (52.4)	850 (71.9)	438 (37.1)	851 (72.0)	599 (50.7)	592 (50.1)
**SSA region of origin**^a^							
West Africa	1446 (56.2)	800 (55.3)	1054 (72.9)	597 (41.3)	1077 (74.5)	755 (52.0)	668 (46.2)
East Africa	124 (4.8)	50 (40.3)	82 (66.1)	48 (38.7)	96 (77.4)	48 (38.7)	45 (36.3)
Central Africa	314 (12.2)	184 (58.6)	251 (79.9)	145 (46.2)	225 (71.7)	176 (56.1)	162 (51.6)
Southern Africa	667 (25.9)	409 (61.3)	500 (75.0)	269 (40.3)	472 (70.8)	303 (45.4)	332 (49.8)
**Marital status**							
Married	1132 (44.0)	648 (57.2)	866 (76.5)	472 (41.7)	821 (72.5)	590 (52.0)	505 (44.6)
Not married^b^	1440 (56.0)	801 (55.6)	1031 (71.6)	595 (41.3)	1058 (73.5)	699 (49.0)	710 (49.3)
**Highest level of education**							
Postgraduate degree	757 (29.4)	406 (53.6)	598 (79.0)	335 (44.3)	567 (74.9)	378 (49.9)	349 (46.1)
Bachelor’s degree	1309 (50.9)	750 (57.3)	955 (73.0)	551 (42.1)	969 (74.0)	707 (54.0)	614 (46.9)
Secondary	448 (17.4)	262 (58.5)	312 (69.6)	158 (35.3)	314 (70.1)	181 (40.4)	234 (52.2)
Primary or less	58 (2.3)	31 (53.5)	32 (55.2)	23 (39.7)	29 (50.0)	23 (39.7)	18 (31.0)
**Employment status**							
Employed/self employed	1890 (73.5)	1095 (57.9)	1428 (75.6)	827 (43.8)	1393 (73.7)	991 (52.4)	872 (46.1)
Unemployed/retired	682 (26.5)	354 (51.9)	469 (68.8)	240 (35.2)	486 (71.3)	298 (43.7)	343 (50.3)
**Religion**							
Christianity	2301 (89.5)	1324 (57.5)	1736 (75.4)	957 (41.6)	1699 (73.8)	1170 (50.9)	1112 (48.0)
Others	271 (10.5)	125 (46.1)	161 (59.4)	110 (40.6)	180 (66.4)	119 (43.9)	103 (38.0)
**Occupation**							
Non-healthcare sector	1771 (68.9)	1017 (57.4)	1314 (74.2)	760 (42.9)	1301 (73.5)	801 (45.0)	908 (51.3)
Healthcare sector	801 (31.1)	432 (53.9)	583 (72.8)	307 (38.3)	578 (72.2)	488 (60.9)	307 (38.3)
Health indicators							
**Smoking status**							
Ex-smoker	160 (6.2)	82 (51.3)	108 (67.5)	66 (41.3)	118 (73.8)	70 (44.0)	63 (39.4)
Current smoker	177 (6.9)	114 (64.4)	132 (74.6)	65 (36.7)	133 (75.1)	75 (42.4)	102 (57.6)
Non-smoker	2235 (86.9)	1253 (56.1)	1657 (74.1)	936 (41.9)	1628 (72.8)	1144 (51.0)	1050 (47.0)
**Any pre-existing condition**							
No	1692 (65.8)	1184 (55.0)	1568 (72.9)	880 (40.9)	1555 (72.3)	1056 (49.0)	1008 (46.9)
Yes	880 (34.2)	265 (63.0)	329 (78 .2)	187 (44.4)	324 (77.0)	233 (55.0)	207 (49.2)
**History of previous vaccination**							
No	1692 (65.8)	910 (53.8)	1229 (72.6)	661 (39.1)	1237 (73.1)	803 (47.0)	793 (46.9)
Yes	880 (34.2)	539 (61.3)	668 (75.9)	406 (46.1)	642 (72.9)	486 (55.0)	422 (47.9)

*HCW* Healthcare workers

^a^Items have some missing responses

^b^Includes widowed, divorced and never married people. Postgraduate degree includes Masters /PhD

Television and social media were the main sources of information for more than two-thirds (*n* = 1897 and 1879, respectively) of the participants in this study during the pandemic, while less than half relied on the newspaper (*n* = 1067, 41.5%) for such information (Table 1). This was consistent across regions, age groups and gender. More than half of the Central African participants reported that they sought COVID-19-related information from HCWs, whereas East African participants relied less on this source of information. Fifty-five percent of those with a pre-existing health condition and those that had previous vaccination reported that they relied on HCWs for COVID-19-related information.

### Percentage of vaccine acceptance, hesitance, and resistance by the information sources

**Fig. 1:**
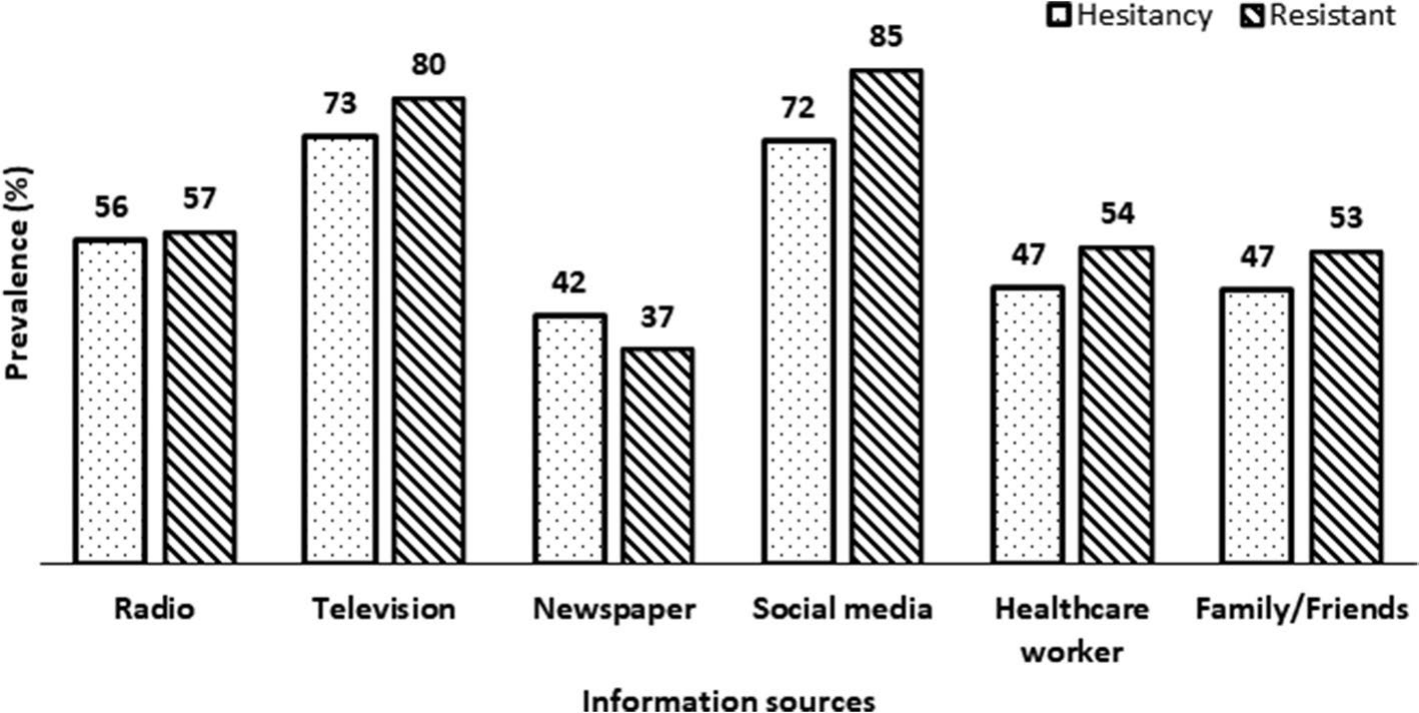
Prevalence of COVID-19 vaccination, hesitancy, resistance by information sources in sub-Saharan Africa, during the pandemic (*n* = 2572)

The proportion of COVID-19 vaccinated, hesitant and resistant participants at the time of this study was 14.9, 17.8, and 67.3%, respectively. Figure 1 displays the proportion of participants who reported COVID-19 hesitancy and resistance, across the different media sources used by the participants during the pandemic. A total of 17% of mainstream listeners and 13% of social media users were vaccinated at the time of this study. Irrespective of the participants’ source of information during the pandemic, the proportion who resisted the vaccine was significantly higher and ranged from 37% among newspaper readers to 85% among social media users. In comparison, the proportion who were hesitant to take the vaccine ranged from 42% among newspaper readers to 73% among those who watched TV during the pandemic.

The Chi-square test found significant associations between the participants’ vaccination status and their reliance on social media (*p* < 0.0001), TV (*p* = 0.004), HCWs (*p* < 0.0001) and friends/families (*p* = 0.001) for COVID-19-related information, during the pandemic.

### Socio-demographic, and health indicators associated with COVID-19-related information sources

The full set of findings from the multinomial logistic regression analyses for the characteristics of those that relied on the various sources of information during the pandemic, after adjusting for the potential cofounders, is presented in Table 2. In this study, reliance on the mainstream media for information during the pandemic was more likely to be observed among Central and Southern African participants, whereas social media was less likely to be used for COVID-19 information retrieval in those with primary education (aORs = 0.36, 95%CI = 0.20, 0.62) and non-Christians (aORs = 0.74, 95%CI = 0.56, 0.97).

**Table 2:** Adjusted odd ratios (AORs) of factors associated with information sources used by participants in sub-Saharan Africa during the pandemic

Variables	Radio	Television	Newspaper	Social media	HCW	Family/Friends
Demography	AORs [95% CI]	AORs [95% CI]	AORs [95% CI]	AORs [95% CI]	AORs [95% CI]	AORs [95% CI]
**Sex**						
Males	Reference	Reference	Reference			Reference
Females	0.72 [0.81, 0.84]	0.81 [0.68, 0.98]	0.73 [0.62, 0.86]	–	–	1.23 [1.05, 1.45]
**SSA region of origin**						
West Africa	Reference	Reference	Reference	Reference	Reference	Reference
East Africa	0.53 [0.37, 0.78]	0.74 [0.50, 1.10]	0.88 [0.60, 1.29]	1.18[0.76, 1.83]	0.56 [0.38, 0.82]	0.66 [0.45, 0.97]
Central Africa	1.16 [0.90, 1.50]	1.69 [1.24, 2.29]	1.20 [0.93, 1.54]	0.92[0.70, 1.22]	1.37 [1.07, 1.77]	1.12 [0.87, 1.44]
Southern Africa	1.49 [1.22, 1.81]	1.44 [1.14, 1.81]	1.11 [0.91, 1.36]	0.89 [0.72, 1.11]	0.89 [0.73, 1.08]	1.03 [0.84, 1.27]
**Highest level of education**						
Postgraduate degree		Reference	Reference	Reference	Reference	Reference
Bachelor’s degree		0.71 [0.57, 0.88]	0.97 [0.81, 1.17]	0.95 [0.77, 1.17]	1.20 [1.00, 1.45]	1.01 [0.84, 1.21]
Secondary		0.53 [0.40, 0.70]	0.73 [0.55, 0.96]	0.82 [0.62, 1.08]	0.86 [0.67, 1.11]	0.96 [0.74, 1.24]
Primary or less		0.34 [0.19, 0.61]	0.96 [0.54, 1.69]	0.36 [0.20, 0.62]	0.83 [0.47, 1.46]	0.44 [0.25, 0.80]
**Employment status**						
Employed/self employed	Reference		Reference			
Unemployed/retired	0.72 [0.60, 0.88]		0.72 [0.59, 0.89]			
**Religion**						
Christianity	Reference	Reference		Reference		
Others	0.57 [0.44, 0.74]	0.45 [0.34, 0.59]		0.74 [0.56, 0.97]		0.65 [0.50, 0.85]
**Occupation**						
Non-healthcare sector	Reference		Reference		Reference	Reference
Healthcare sector	0.82 [0.69, 0.99]		0.71 [0.59, 0.86]		1.81 [1.51, 2.17]	0.58 [0.48, 0.69]
**Smoking status**						
Ex-smoker						Reference
Current smoker						1.97 [1.26, 3.10]
Non-smoker						1.35 [0.96, 1.89]

Confidence intervals (CI) that does not include 1.00 are significant variables

Postgraduate degree includes Masters /PhD

*HCW* Healthcare workers

Central African participants and those who worked in health sectors were more likely to rely on HCWs for COVID-19-related information as compared to West African participants and those who worked in non-healthcare sectors, during the pandemic. Compared with males, female participants were less likely to listen to the radio, watch TV and read the newspaper but more likely to rely on friends and family (aOR = 1.23, 95%CI = 1.05, 1.45), for COVID-19-related information, during the pandemic. Current smokers were also more likely to rely on friends and family (aOR = 1.97, 95%CI = 1.26, 3.10), while those with primary or no education as well as non-Christians were less likely to rely on social media for information, during the pandemic.

### Associations between COVID-19 vaccine hesitancy, resistance, and sources of information used by participants in SSA during the pandemic

The aORs and their 95%CI for factors associated with vaccine hesitancy and vaccine resistance are presented in Tables 3 and 4, respectively. After adjusting for the potential confounders, in this study, participants who listened to the radio, those who watched TV, and social media users, during the pandemic, were less likely to report COVID-19 vaccine hesitancy. As shown in Table 4, age (29-38 years), SSA region of origin (East Africa), educational level (primary education or less), religion and occupation of the participants were associated with resistance towards COVID-19 vaccination. Except for those who listened to the radio, reliance on other media sources for COVID-19-related information was significantly associated with vaccine resistance, with the strongest association found among social media users (aOR = 2.13 95%CI = 1.62, 2.80) Table 4. Also, those who watched TV and people who relied on HCWs and friends/family for COVID-19-related information were more likely to resist COVID-19 vaccination, whereas reading the newspaper reduced the likelihood of vaccine hesitancy (aOR = 0.77, 95%CI 0.62, 0.95) among the participants.

**Table 3:** Adjusted odd ratios for factors associated with media sources and vaccine hesitancy among participants in sub-Saharan Africa during the pandemic

Variables	Total	Radio	TV	Newspaper	Social media	HCWs	Family/Friends
Demography	AORs [95% CI]	AORs [95% CI]	AORs [95% CI]	AORs [95% CI]	AORs [95% CI]	AORs [95% CI]	AORs [95% CI]
**Age category in years**							
18–28	Reference	Reference	Reference	Reference	Reference	Reference	Reference
29–38	0.85 [0.66, 1.10]	0.86 [0.66, 1.11]	0.85 [0.66, 1.10]	0.85 [0.66, 1.10]	0.85 [0.66, 1.10]	0.84[0.65, 1.09]	0.85[0.66, 1.10]
39–48	0.88 [0.64, 1.19]	0.88 [0.65, 1.20]	0.88 [0.67, 1.99)	0.88 [0.64, 1.19]	0.87[0.64, 1.19]	0.88[0.65, 1.20]	0.88 [0.65, 1.20]
49+	0.86 [0.61, 1.20]	0.86 [0.61, 1.21]	0.86 [0.61, 1.21]	0.85 [0.60, 1.19]	0.86[0.61, 1.21]	0.86 [0.61, 1.21]	0.86 [0.61, 1.21]
**Sex**							
Males	Reference	Reference	Reference	Reference	Reference	Reference	Reference
Females	0.83 [0.70, 0.99]	0.82 [0.69, 0.98]	0.83 [0.69, 0.99]	0.84 [0.70, 0.99]	0.83[0.70, 0.99]	0.84 [0.70, 0.99]	0.84 [0.70, 1.00]
**SSA Region of Origin**							
West Africa	Reference	Reference	Reference	Reference	Reference	Reference	Reference
East Africa	1.10 [0.73, 1.64]	1.07 [0.71, 1.60]	1.08 [0.72, 1.62]	1.10[0.73, 1.64]	1.10[0.74, 1.65]	1.06 [0.71, 1.58]	1.08[0.72, 1.62]
Central Africa	0.86 [0.66, 1.13]	0.87 [0.66, 1.13]	0.88 [0.67, 1.15]	0.86 [0.66, 1.12]	0.86 [0.66, 1.12]	0.88[0.68, 1.16]	0.87 [0.66, 1.13]
Southern Africa	1.24 [0.98, 1.56]	1.26 [1.00, 1.59]	1.26 [1.00, 1.58]	1.23[0.98, 1.55]	1.23 [0.97, 1.54]	1.23[0.98, 1.55]	1.24 [0.98, 1.56]
**Marital Status**							
Married	Reference	Reference	Reference	Reference	Reference	Reference	Reference
Not married	0.73 [0.58, 0.90]	0.73 [0.58, 0.90]	0.73 [0.58, 0.90]	0.72 [0.58, 0.90]	0.73[0.59, 0.91]	0.73 [0.58, 0.90]	0.73 [0.59, 0.91]
**Highest level of education**							
Postgraduate Degree	Reference	Reference	Reference	Reference	Reference	Reference	Reference
Bachelor’s degree	0.89 [0.72, 1.10]	0.90 [0.73, 1.11]	0.88 [0.71, 1.09]	0.89[0.72, 1.10]	0.89 [0.72, 1.09]	0.90[0.73, 1.12]	0.89 [0.72, 1.10]
Secondary	0.84 [0.61, 1.16]	0.85 [0.62, 1.18]	0.83 [0.60, 1.14]	0.85[0.61, 1.17]	0.83 [0.60, 1.44]	0.84 [0.61, 1.16]	0.84 [0.61, 1.16]
Primary or less	0.59 [0.32, 1.12]	0.61 [0.32, 1.14]	0.57 [0.30, 1.07]	0.59 [0.32, 1.12]	0.56[0.30, 1.06]	0.58[0.31, 1.10]	0.58 [0.31, 1.09]
**Employment status**							
Employed/self employed	Reference	Reference	Reference	Reference	Reference	Reference	Reference
Unemployed/retired	1.28 [1.00, 1.63]	1.26 [0.99, 1.61]	1.26 [0.99, 1.61]	1.28 [1.01, 1.64]	1.28 [1.00, 1.63]	1.27 [0.99, 1.61]	1.28 [1.00, 1.63]
**Religion**							
Christianity	Reference	Reference	Reference	Reference	Reference	Reference	Reference
Others	1.29 [0.96, 1.73]	1.26 [0.94, 1.69]	1.24 [0.93, 1.67]	1.29 [0.96, 1.73]	1.27[0.95, 1.71]	1.28 [0.95, 1.71]	1.28 [0.95, 1.71]
**Occupation**							
Non-healthcare sector	Reference	Reference	Reference	Reference	Reference	Reference	Reference
Healthcare sector	0.59 [0.48, 0.72]	0.58 [0.48, 0.71]	0.58 [0.48, 0.71]	0.59 [0.48, 0.72]	0.58[0.48, 0.71]	0.61[0.50, 0.75]	0.58 [0.47, 0.71]
**Smoking status**							
Ex-smoker	Reference	Reference	Reference	Reference	Reference	Reference	Reference
Current smoker	0.88 [0.54, 1.42]	0.90 [0.55, 1.45]	0.90 [0.55, 1.45]	0.88 [0.54, 1.42]	0.89 [0.55, 1.43]	0.88[0.54, 1.42]	0.90 [0.56, 1.45]
Non-smoker	1.04 [0.73, 1.50]	1.06 [0.74, 1.52]	1.07 [0.74, 1.53]	1.04 [0.72, 1.49]	1.04[0.73, 1.50]	1.06[0.74, 1.53]	1.05[0.73, 1.52]
**Any pre-existing condition**							
No	Reference	Reference	Reference	Reference	Reference	Reference	Reference
Yes	0.81 [0.64, 1.03]	0.82 [0.65, 1.04]	0.82 [0.64, 1.04]	0.81 [0.64, 1.03]	0.82 [0.64, 1.04]	0.82[0.65, 1.05]	0.81[0.64, 1.03]
**Previous vaccine as a child**							
No	Reference	Reference	Reference	Reference	Reference	Reference	Reference
Yes	0.90 [0.75, 1.08]	0.91 [0.76, 1.09]	0.90 [0.75, 1.08]	0.84 [0.75, 1.07]	0.90 [0.75, 1.08]	0.91[0.76, 1.10]	0.90 [0.75, 1.08]

Confidence intervals (CI) that does not include 1.00 are significant variables

Postgraduate degree includes Masters /PhD

*HCW* Healthcare workers

**Table 4:** Adjusted odd ratios for factors associated with media sources and vaccine resistance among participants in sub-Saharan Africa during the pandemic

Variables	Total	Radio	TV	Newspaper	Social media	HCWs	Family/Friends
Demography	AORs [95% CI]	AORs [95% CI]	AORs [95% CI]	AORs [95% CI]	AORs [95% CI]	AORs [95% CI]	AORs [95% CI]
Age category in years							
18-28	Reference	Reference	Reference	Reference	Reference	Reference	Reference
29-38	1.58 [1.16, 2.15]	1.58 [1.16, 2.15]	1.58 [1.16, 2.15]	1.59 [1.17, 2.17]	1.60 [1.17, 2.19]	1.59 [1.17, 2.17]	1.58 [1.16, 2.15]
39-48	1.13 [0.78, 1.66]	1.13 [0.77, 1.66]	1.13 [0.77, 1.65]	1.15 [0.78, 1.68]	1.15 [0.78, 1.68]	1.13 [0.77, 1.65]	1.13 [0.77, 1.66]
49+	1.30 [0.86, 1.96]	1.30 [0.86, 1.96]	1.29 [0.85,1.95]	1.34 [0.89, 2.04]	1.29 [0.85, 1.95]	1.30 [0.86, 1.97]	1.29 [0.85, 1.96]
Sex							
Males	Reference	Reference	Reference	Reference	Reference	Reference	Reference
Females	1.11[0.89, 1.37]	1.11 [0.90, 1.37]	1.12 [0.91, 1.39]	1.09 [0.88, 1.35]	1.12 [0.90, 1.38]	1.10[0.89, 1.37]	1.09 [0.88, 1.35]
SSA Region of Origin							
West Africa	Reference	Reference	Reference	Reference	Reference	Reference	Reference
East Africa	1.65 [1.07, 2.53]	1.65[1.07, 2.54]	1.69 [1.10, 2.59]	1.64 [1.07, 2.53]	1.63 [1.06, 2.51]	1.71[1.11, 2.63]	1.70 [1.10, 2.61]
Central Africa	0.73 [0.52, 1.04]	0.73 [0.52, 1.04]	0.72 [0.51, 1.02]	0.74 [0.52, 1.05]	0.75 [0.53, 1.07]	0.72 [0.51, 1.02]	0.73 [0.51, 1.03]
Southern Africa	1.02 [0.77, 1.33]	1.01[0.77, 1.33]	0.99 [0.75, 1.31]	1.03 [0.78, 1.35]	1.05 [0.79, 1.38]	1.02[0.78, 1.32]	1.01 [0.77, 1.33]
Marital Status							
Married	Reference	Reference	Reference	Reference	Reference	Reference	Reference
Not married	1.20 [0.92, 1.55]	1.19[0.92, 1.55]	1.20 [0.92, 1.56]	1.22 [0.94, 1.59]	1.17 [0.90, 1.52]	1.19 [0.91, 1.55]	1.19[0.91, 155]
Highest level of education							
Postgraduate Degree	Reference	Reference	Reference	Reference	Reference	Reference	Reference
Bachelor’s degree	0.86 [0.67, 1.11]	0.86[0.67, 1.11]	0.88 [0.68, 1.13]	0.86[0.67, 1.11]	0.87[0.68, 1.13]	0.85[0.66, 1.10]	0.87 [0.67, 1.11]
Secondary	0.86 [0.58, 1.26]	0.86 [0.58, 1.26]	0.88 [0.60, 1.30]	0.84[0.58, 1.24]	0.89[0.61, 1.32]	0.86[0.59, 1.26]	0.86 [0.58, 1.26]
Primary or less	0.27 [0.08, 0.91]	0.27[0.08, 0.91]	0.29 [0.09, 0.98]	0.27[0.08, 0.91]	0.30[0.09, 1.02]	0.28 [0.08, 0.92]	0.28[0.09, 0.95]
Employment status							
Employed/self employed	Reference	Reference	Reference	Reference	Reference	Reference	Reference
Unemployed/retired	0.84 [0.63, 1.13]	0.84[0.63, 1.13]	0.85 [0.64, 1.14]	0.83 [0.62, 1.11]	0.85 [0.63, 1.14]	0.85 [0.63, 1.14]	0.84[0.63, 1.13]
Religion							
Christianity	Reference	Reference	Reference	Reference	Reference	Reference	Reference
Others	0.57 [0.38, 0.84]	0.57[0.38, 0.84]	0.60 [0.40, 0.89]	0.56[0.38, 0.84]	0.60 [0.40, 0.88]	0.58 [0.39, 0.85]	0.59 [0.40, 0.87]
Occupation							
Non-healthcare sector	Reference	Reference	Reference	Reference	Reference	Reference	Reference
Healthcare sector	0.64 [0.50, 0.82]	0.64[0.50, 0.82]	0.65 [0.51, 0.83]	0.63[0.49, 0.81]	0.65[0.51, 0.83]	0.62 [0.48, 0.79]	0.66 [0.52, 0.85]
Smoking status							
Ex-smoker	Reference	Reference	Reference	Reference	Reference	Reference	Reference
Current smoker	1.65 [0.92, 2.96]	1.65 [0.92, 2.96]	1.61 [0.90, 2.90]	1.64 [0.91, 2.94]	1.62 [0.90, 2.91]	1.65 [0.92, 2.96]	1.58 [0.88, 2.83]
Non smoker	1.29 [0.81, 2.05]	1.29 [0.81, 2.04]	1.25 [0.79, 1.99]	1.31 [0.82, 2.07]	1.30 [0.82, 2.06]	1.27 [0.80, 2.01]	1.26[0.79, 2.00]
Any pre-existing condition							
No	Reference	Reference	Reference	Reference	Reference	Reference	Reference
Yes	0.97 [0.72, 1.30]	0.97 [0.72, 1.30]	0.95 [0.71, 1.28]	0.97 [0.72, 1.31]	0.93[0.69, 1.26]	0.95[0.71, 1.28]	0.96 [0.71, 1.29]
Previous vaccine as a child							
No	Reference	Reference	Reference	Reference	Reference	Reference	Reference
Yes	0.82 [0.66, 1.03]	0.82[0.66, 1.03]	0.82 [0.65, 1.02]	0.84 [0.67, 1.05]	0.82 [0.65, 1.03]	0.81 [0.64, 1.01]	0.82 [0.65, 1.03]

Confidence intervals (CI) that does not include 1.00 are significant variables

Postgraduate degree includes Masters /PhD

*HCW* Healthcare workers

**Fig. 2:**
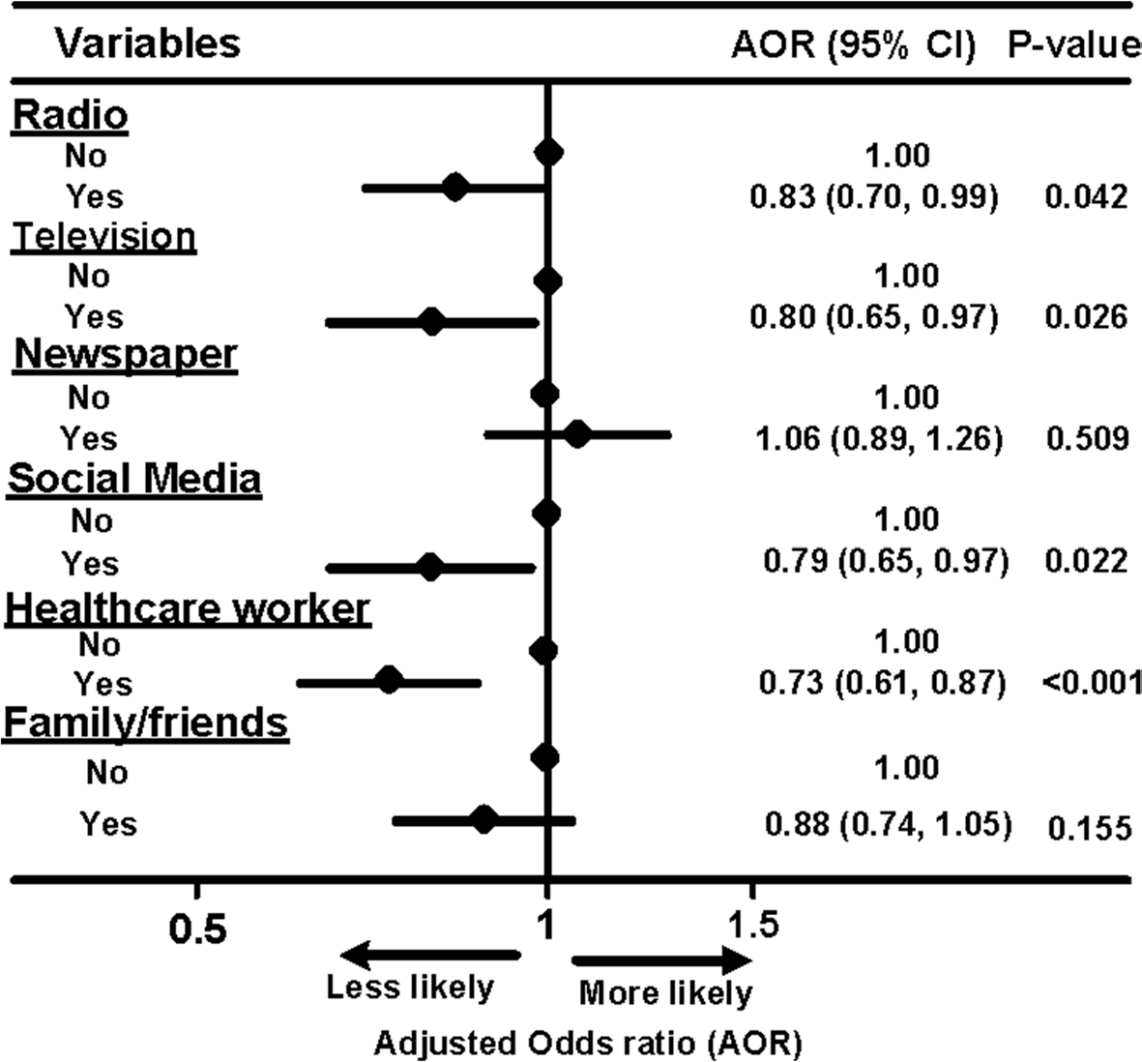
Forest plot of association between main information sources and vaccine hesitancy and resistance among the participants in sub-Saharan Africa, during the pandemic

**Fig. 3:**
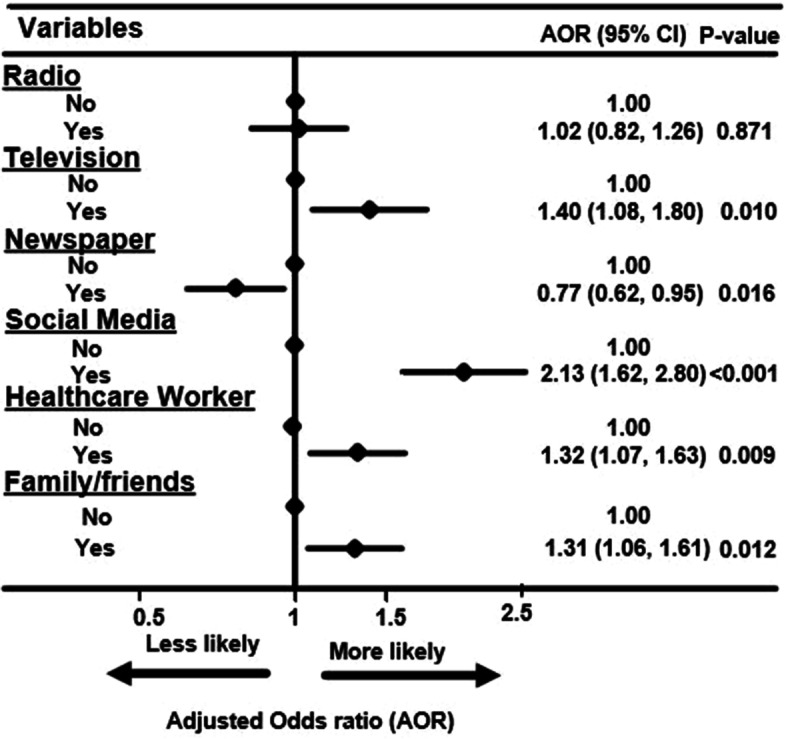
Forest plot of association between main information sources and vaccine resistance among the participants in sub-Saharan Africa, during the pandemic

The forest plots showing the adjusted odd ratios for the association between the media sources used by the participants in SSA countries during the pandemic and vaccine hesitancy and resistance are shown in Figs. 2 and 3, respectively. Figure 2 shows that COVID-19 vaccine hesitancy was significantly associated with four of the six media sources examined in this study. Reliance on HCWs, social media and traditional sources (TV and radio) for COVID-19-related information during the pandemic reduced the odds of COVID-19 vaccine hesitancy by 27, 21, 20 and 17%, respectively.

There was a strong association between the use of social media and resistance towards COVID-19 vaccination (aOR = 2.13, 95%CI 1.62, 2.80) as seen in Fig. 3. Other factors such as watching TV and reliance on friends/families for information related to COVID-19 were also associated with COVID-19 vaccine resistance among the participants. Those who relied on the newspapers for information during the pandemic were less likely to be resistant towards taking the COVID-19 vaccines compared to those who did not (Fig. 3).

## Discussion

This study was undertaken to determine the role of different information sources on COVID-19 vaccine hesitancy and resistance in SSA. Consistent across age groups, gender and regions, television and Facebook, were the main sources of up-to-date information for participants in SSA during the pandemic. However, information from these sources, particularly those obtained from social media platforms, can be misleading, and as shown in the present study, social media users were twice more likely to resist the COVID-19 vaccines compared with non-users. Those who relied on the TV, HCWs, friends, and family members for their up-to-date information had a higher likelihood of vaccine resistance than their counterparts. In contrast, the odds for vaccine resistance were significantly reduced among those who reported that the newspaper was their main source of information during the pandemic.

Although the finding of a strong independent association between social media use and vaccine resistance was contrary to previous studies on smaller samples in Saudi Arabia [51, 52], this is important considering the wide utilisation of Facebook as the main source of information by many participants during the pandemic. A Facebook IQ survey revealed that more than 95 million people in SSAs access Facebook, with 97% of these doing so on handheld and mobile devices each month. Therefore, these popular sources of information (Television and Facebook) must be used to convey reliable, science-based information about COVID-19 vaccines and future pandemics to the millions of SSA people.

Smokers and females were more likely to rely on family and friends for COVID-19-related information, but less likely to rely on mainstream media (such as TV) than their male counterparts. There was a lower likelihood for non-Christians and those with lower education to rely on social media for information during the lockdown. Of the information sources, reliance on social media showed the strongest association with COVID vaccine hesitancy and resistance. After adjusting for potential covariates, information sources played a significant role in vaccine hesitancy and resistance among SSAs. Those who relied on information obtained from watching TV and family/friends were more likely to resist the COVID vaccine when compared to those who did not rely on those media sources. Listening to the radio and obtaining information from HCWs had a positive influence on intent towards vaccination because it reduced their likelihood of being resistant and hesitant towards COVID-19 vaccination. The negative influence of TV and social media use on COVID-19 vaccination reported in this study was not surprising as some emerging anti-vaccine television and social media campaigns are responsible for generating and perpetuating vaccine hesitancy and resistance. The high prevalence of inaccurate and negative information on social media regarding COVID-19 may predict a greater likelihood of negative vaccine intent in this case as well [53, 54]. In addition, social media is generally unregulated and has enabled people with anti-vaccine beliefs to generate and disseminate information freely [55]. The findings of this study are consistent with a previous study which found that, relative to social media and the internet, there was a positive association between reliance on traditional news sources and intention to uptake a COVID-19 vaccine in the United States [56]. Another previous work also highlighted the role of negative information on social media in shaping individual perceptions regarding human papillomavirus (HPV) vaccination intent [57].

Central and Southern African participants showed greater reliance on mainstream media for COVID-19-related information, particularly watching TV, and this increased their likelihood of not taking the vaccine. This finding could, in part, be related to the nature of lockdowns in different sub-Saharan countries. For instance, South Africa went into Level 5 (hard lockdown) quite early in the pandemic (March 2020), and residents were mostly confined to their homes, watching TV [58]. Reliance on social media platforms for COVID-19-related information was associated with higher educational levels, which agreed with a study from South Africa [58] which found that education-related inequalities were visible in the use of COVID-19 preventive measures in South Africa.

The finding that the participants with pre-existing medical conditions or those who had a prior history of vaccinations were more reliant on HCWs for COVID-19-related information during the pandemic suggests that HCWs are trusted to have a better understanding of COVID-19 information, and as such, they can be a source of essential care and information in future pandemics. In a previous study, participants rated health information from doctors and other health workers as highly reliable [59]. This assertion is supported by a recent study that showed that HCWs are essential front liners, working to ensure the health of older adults and those with chronic conditions or disabilities during the COVID-19 pandemic [60]. The high vaccination and low hesitancy rates reported among participants who relied on HCWs for information were consistent with a previous study, which showed that HCWs have adequate information on vaccines and have the ability and confidence to communicate such information effectively [61]. This finding supports the idea that HCWs, can positively influence the use of vaccines and have the potential to impact COVID-19 vaccination in SSA. However, recent literature has also warned of the inadequate capacity of HCWs to deal with anti-vaccine messages on social media [62].

One interesting finding of this paper is the resistant effect of information derived from HCW reported by participants. Studies among Africans have shown that HCWs themselves are resistant to the vaccine with their information being obtained from unreliable sources such as social media, friends and family [63, 64]. Safety concerns, insufficient or inaccurate information, lack of trust in the government’s capacity to manage, and personal beliefs are factors that have been reported to influence the acceptance or resistance of HCWs to the vaccine [65–67]. The likelihood of such health workers passing on information to the populace with content that may be tainted with their own beliefs and inaccuracies can contribute to making those who interact with them resistant to the vaccine.

Females were less likely to listen to the radio, watch TV and read newspapers but more likely to rely on friends and family, and this increased their likelihood of vaccine hesitancy. This finding may suggest that women expressed interest in COVID-19 issues with their friends and family (leaving very little room for individual proactive decision-making) while men were significantly more likely than women to get such information from the radio, TV and newspapers. The study also showed differences in behaviour, such that the less educated, non-Christians were not more reliant on social media platforms for information during the pandemic than their counterparts. For those who were more likely to be resistant (such as those who watched TV and those who relied on their families and friends for information), additional vaccine promotional efforts would be required.

### Limitations and strengths

Some limitations should be considered when interpreting the findings of this study. First, this was a cross-sectional study, and as such, we cannot determine causation. Second, like previous studies conducted during COVID-19 in SSA [34, 47, 68, 69], we utilized an internet-based methodology which was the only reliable means to disseminate information at the time of this study. The survey was distributed electronically using social media platforms and emails because it was difficult to physically access some participants in some places due to the protective measures still in place at the time of the study. This method of soliciting participants may have inadvertently excluded some potential participants whose opinions differed, such as those without internet access and people living in rural areas, where internet penetration remains relatively low [70]. Third, the survey was presented in English and French and thus inadvertently excluding non-English and non-French speaking countries in SSA from participating. Fourth, although the study showed satisfactory internal validity, its generalization or transferability to all SSA countries may be limited. Notwithstanding these limitations, this was the first study from the SSA region to provide insight into some of the impacts of information sources on the acceptance of COVID-19 vaccines which has been a worry to the international community. Although this topic is commonplace as reliance on online information sources is expected to happen during pandemics, no study has demonstrated the impacts of these sources of information on COVID vaccination in the way the present study did, including the use of a robust analysis to control for potential confounders during the analysis and reduce the possibility of a bias. This makes our study a unique one since it provided the first documented evidence from SSA showing the impacts of the lockdown on the behaviour of ordinary citizens.

### Implications of our findings

This study provides an understanding of how the exposure of SSAs to various media sources during the pandemic, influences their attitude toward the COVID-19 vaccination program. Our focus on COVID-19 vaccine hesitancy and resistance is important because of the need to stem the pandemic by vaccinating enough people in the face of the recent rise in infections [11]. The findings are important because people’s negative attitudes toward vaccination in general, and their hesitancy or resistance to the COVID-19 vaccine, is a growing public health problem. This study provides insight into how the various media outlets commonly used by the participants living in different SSAs regions to obtain COVID-19-related information affect their attitude towards vaccine uptake. This finding underlines the importance of media exposure, suggesting that the media can be used to improve vaccine literacy across the region [71]. In addition, this study contributes to our understanding of the interplay between SSA regions and media exposure during the pandemic. For example, the study found greater reliance on the mainstream media for COVID-19-related information among those from Central and Southern Africa, which negatively influenced vaccine uptake. This insight has important practical implications by informing us about the dynamics of individuals’ attitudes and would help researchers understand the underlying factors that influence the acceptance of vaccination during a pandemic. This study will help public health and health promotion officers in various SSA countries design more effective communications and interventions.

Furthermore, the very low vaccination rate observed in this study raises the concern of vaccine nationalism with challenges of vaccine inequity in low and middle-income countries which was shown to be counterproductive during the pandemic [5, 12, 72]. High-income countries prioritized investment in the stock of vaccinations over immediate capacity building and delivery of such life-saving vaccines by healthcare systems. These lessons are important in tackling future pandemics. Although vaccinations are the only effective means of tackling viral diseases, prior studies have demonstrated that many people do not believe in their safety and effectiveness [14]. There is also the possibility that previously eradicated infections may re-emerge in some regions. People need to be educated about vaccines, their safety and their efficacy. The media can be used to boost people’s confidence in taking the vaccine [14, 73, 74].

## Conclusions

The findings of this study suggest that healthcare organizations and governments of SSA fight misinformation by providing factual messages countries need to utilise social media platforms, television, and healthcare workers to provide reliable information to influence vaccine hesitancy and encourage uptake of the COVID-19 vaccination. Failure to access and apply reliable healthcare information, whether for the public or health workers, has always been a major cause of avoidable deaths. More research and investment are needed to improve the availability of reliable healthcare information, protect people from misinformation, and empower people with education on how to identify misinformation. The ongoing trajectory of misinformation - from vaccine hesitancy to previous infectious diseases to COVID-19 –calls for global action as the ‘infodemic’ of the next public health emergency may be worse than the current COVID infodemic.

## Supplementary Information

**Additional file 1: Supplementary Table S1.** Sample of Survey Item.

**Additional file 2: Figure S1.** Country of origin of respondents.

## Data Availability

The dataset supporting the conclusions of this article is included within the article (and its additional files). Data is also available on request from the corresponding author OUL.

## Abbreviations

COVID-19: Coronavirus disease
SSA: Sub–Saharan Africa
OR: Odds ratio
AOR: Adjusted odds ratio
CI: Confidence interval
TV: Television
HCW: Healthcare worker
WHO: World Health Organization
