# Supplementary material for: The impact of information sources on COVID-19 vaccine hesitancy and resistance in sub-Saharan Africa
Source: BMC Public Health. 2023 Jan 6;23:38. doi: 10.1186/s12889-022-14972-2 (PMC9816548; doi:10.1186/s12889-022-14972-2)
Supplement: Supplementary file 1 — Additional file 1: Supplementary Table S1. Sample of Survey Item. [file 12889_2022_14972_MOESM1_ESM.docx]

**Supplementary Table S1 Sample of Survey Item**

Do you understand the above and consent to willingly take part in this survey? Yes/No

1. Country of origin
2. Province/State/County (give the state where you have been working in the past 6-12 months)
3. Country of residence
4. Gender

- Male
- Female
- Others

1. Age (Years)
2. Marital Status

- Single
- Married/de-facto
- Separated/divorced
- Widowed

1. Religion

- Islam
- Christian
- African Traditional religion
- Others

1. Highest level of Education

- No formal education
- Primary Education
- Secondary Education (including High school)
- Polytechnic/diploma
- University degree (bachelors/professional)
- Postgraduate degree (Masters/Phd)

1. Employment Status (Tick the ones that apply to you, you can choose more than one option)

- I am a paid employee
- I am self-employed
- I am unemployed or retired

1. What is your Occupation (you can choose the options that apply)

- Health care worker/professional (Doctor, Nurse, Pharmacist, Community Health and Extension worker, Age care worker, disability service worker, medical laboratory scientist etc)
- Non Health care worker
- Student (health care related course/ non health care related course)

1. Tell us about your smoking status (*Choose the correct option*)
   1. Previous smoker
   2. Current smoker
   3. Never smoked
2. Do you have any of the following conditions (Tick the option(s) that applies to you)
   1. Cancer
   2. Kidney disease
   3. Any heart condition
   4. Obesity
   5. Pregnant
   6. Diabetes
   7. Sickle cell disease
   8. Hypertension
   9. Asthma
   10. Other, specify ………………………………..
3. Have you been ever vaccinated for any disease before?

Yes/No

1. Which condition were you vaccinated against? You can select more than one option
   1. Tuberculosis
   2. MMR (measles, mumps and Rubella)
   3. Yellow fever
   4. Polio
   5. Diphtheria, pertussis, and tetanus (DPT)
   6. Flu Influenza
   7. Whooping cough
   8. BCG vaccine
   9. Chickenpox (Varicella) Vaccine
   10. Hepatitis
   11. Others

**Information sources**

1. Where do you get most of the information on COVID-19 vaccine? Tick all that applies
   1. Radio Yes/No
   2. Television Yes/No
   3. Newspaper Yes/No
   4. Social media (such as Facebook, WhatsApp, Twitter) Yes/No
   5. health care workers Yes/No
   6. family and friends Yes/No
   7. Other sources (please specify)………………….……

**PERCEPTION OF COVID-19 VACCINE**

1. Have you been vaccinated against COVID-19? Yes/No
2. If a COVID-19 vaccine is available in your country, will you be willing to be vaccinated against the disease? Yes/No
3. If no, are you more likely to accept the COVID-19 vaccine (***You can select more than one option)***
   1. If financial incentives are giving to parents
   2. If Monetary rewards are given to health care providers involved in vaccination
   3. If it is given for free
   4. If there is adequate information regarding the specific vaccine
   5. If I can get more education on the vaccine, their side effects and how effective they are
   6. If it is a travel condition
   7. If it is an employment condition
   8. If many people start receiving the vaccine
   9. If I get positive feedback from those who have been vaccinated
4. If No, which of the following factors contribute to your decision to not accept a COVID-19 vaccine? ***You can select more than one option***

- Advice from religious leaders
- Advice from politicians
- Mistrust for the pharmaceutical company
- Mistrust of the health system in my country
- Mistrust in the medical process for developing the vaccine
- Mistrust for the country where the vaccine was produced
- Personal belief or past historical experiences with vaccines
- Concerned about safety of the COVID-19 vaccine
- Not enough information from healthcare providers
- Information from the media

**THANK YOU FOR TAKING OUR SURVEY**
