# Supplementary material for: The impact of information sources on COVID-19 vaccine hesitancy and resistance in sub-Saharan Africa
Source: BMC Public Health. 2023 Jan 6;23:38. doi: 10.1186/s12889-022-14972-2 (PMC9816548; doi:10.1186/s12889-022-14972-2)
Supplement: Supplementary file 2 — Additional file 2: Figure S1. Country of origin of respondents. [file 12889_2022_14972_MOESM2_ESM.docx]

Figure S1: Country of origin of respondents
